# Dual Beneficial Effects of Methylnissolin-3-O-β-d-Glucopyranoside on Obesity-Induced Inflammatory Responses in Adipocyte-Macrophage Co-Culture

**DOI:** 10.3390/plants11131715

**Published:** 2022-06-28

**Authors:** Dahae Lee, Xiaohua Wu, Ingo Lange, Shugeng Cao, Ki Sung Kang

**Affiliations:** 1 College of Korean Medicine, Gachon University, Seongnam 13120, Korea; pjsldh@gachon.ac.kr; 2 Department of Pharmaceutical Sciences, Daniel K. Inouye College of Pharmacy, University of Hawai’i at Hilo, Hilo, HI 96720, USA; xiaohua3@hawaii.edu (X.W.); ingo@hawaii.edu (I.L.)

**Keywords:** adipocytes, macrophage, adipogenesis, inflammation

## Abstract

Methylnissolin-3-O-β-d-glucopyranoside (MNG) is a pterocarpan analog, which protects EA.hy926 cells against oxidative damage through the Nrf2/HO-1 pathway. However, the effects of MNG on obesity-induced inflammatory responses in adipocyte-macrophage co-culture remain unclear. A differentiated murine preadipocyte cell line (3T3-L1) was co-cultured with a murine macrophage cell line (RAW264.7). Intracellular lipid accumulation was determined using Oil Red O staining. Western blotting was performed to investigate the expression of adipogenesis- and inflammation-associated proteins. Cell culture supernatants were assayed using ELISA kits to measure the levels of proinflammatory cytokines such as interleukin 6 (IL-6) and monocyte chemoattractant protein-1 (MCP-1). MNG inhibited lipid accumulation and the production of IL-6 and MCP-1 in the 3T3-L1 and RAW264.7 cell co-culture. Moreover, MNG inhibited the protein expression of CCAAT/enhancer-binding protein alpha (C/EBPα), C/EBPβ, peroxisome proliferator-activated receptor γ (PPARγ), cyclooxygenase 2 (COX-2), and inducible nitric oxide synthase (iNOS) under the same co-culture conditions. MNG also inhibited IL-6 and MCP-1 production compared with the co-culture control. These findings demonstrate that MNG inhibited lipid accumulation and inflammatory response by downregulating IL-6 and MCP-1 production and protein expression of C/EBPβ, C/EBPα, PPARγ, COX-2, and iNOS in co-culture conditions with 3T3-L1 and RAW264.7 cells. These results suggest that MNG may be beneficial in preventing obesity-related inflammatory status.

## 1. Introduction

Obesity is the state of low-grade systemic inflammation closely associated with an increased prevalence of metabolic diseases such as, type 2 diabetes, arteriosclerosis, cardiovascular disease, and cancer [1]. Adipose tissue can recruit and stimulate macrophages by secreting excess proinflammatory cytokines, monocyte chemotactic protein-1 (MCP-1), and free fatty acids [2]. The accumulation of macrophages in adipose tissue can result in the secretion of proinflammatory cytokines, such as interleukin 6 (IL-6), and leads to inflammatory response and obesity-induced insulin resistance in adipose tissue [3]. Therefore, reducing obesity-induced inflammation might attenuate obesity-related diseases. Red ginseng-derived saponin fraction, 6-gingerol (found in ginger), and lunasin (identified in several grains) inhibit obesity-induced inflammatory responses by inhibiting the production of inflammatory mediators MCP-1 and IL-6 in co-culture conditions with 3T3-L1 and RAW264.7 cells [4,5,6]. However, studies of the natural products used to inhibit obesity-induced inflammatory responses and their application to obesity-related inflammatory diseases remain lacking.

Methylnissolin-3-O-β-d-glucopyranoside (MNG) is a natural product of *Astragalus membranaceus* [7]. *Astragalus membranaceus* has been investigated extensively, but MNG as a single chemical entity has been rarely studied regarding its biological activities, except for Nrf2 activation, which we reported in 2020 [7] and 2021 [8]. MNG enhanced the expression of Nrf2, increased Nrf2 nuclear translocation, activated Nrf2 target proteins, and regulated cytoprotective responses to stress caused by reactive oxygen species. In addition, MNG demonstrated effects on the PI3K/Akt pathway involved in controlling Nrf2-ARE (Nrf2 antioxidant response element) activity [8]. However, the activity of MNG against obesity-induced inflammatory responses in adipocyte-macrophage co-culture has not been investigated. Therefore, the effects of MNG on obesity-induced inflammatory responses and its potential molecular mechanism in the co-culture of 3T3-L1 and RAW264.7 cells were investigated in this study.

## 2. Results

### 2.1. Identification of MNG

**Figure 1:**
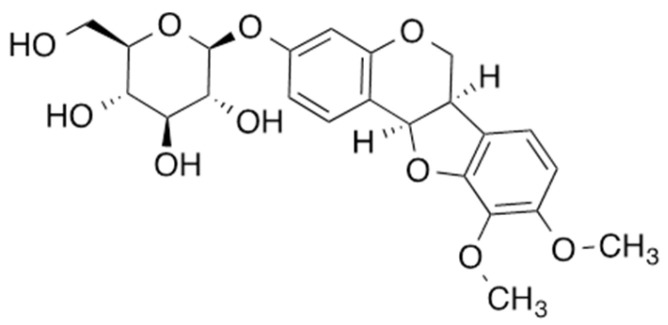
Chemical structure of MNG.

MNG was isolated as a powder with a molecular formula of C_23_H_26_O_10_, which was determined by HRESIMS, requiring 11 degrees of unsaturation. A comprehensive analysis of the 1D and 2D NMR spectra indicated the presence of five aromatic protons, two methoxy groups, seven methines, and two methylene groups. Four spin systems, including a 1,2,3,4-tetrasubstitued benzene ring, a 1,2,4-trisubstitued benzene ring, pyranose, and CH-CH-CH2, were identified in the COSY spectrum. The spin systems were connected based on HMBC correlations, and the compound was identified as MNG (Figure 1) [7,8].

### 2.2. Inhibitory Effects of MNG on Lipid Accumulation and Production of IL-6 and MCP-1 in Co-Culture Condition with 3T3-L1 and RAW264.7 Cells

**Figure 2:**
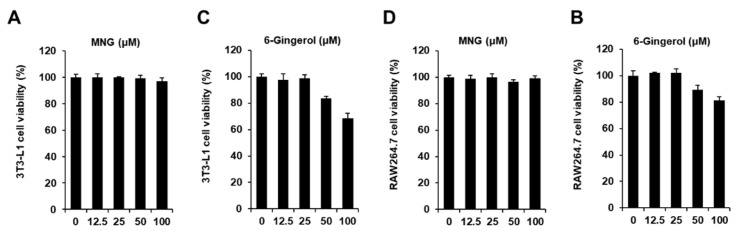
Effect of MNG on the viability of 3T3-L1 preadipocytes and RAW264.7 cells. Effect of (**A**) MNG and (**B**) 6-gingerol (positive control) compared with the control (untreated, 0 μM) on the viability of 3T3-L1 preadipocytes for 24 h. Effect of (**C**) MNG and (**D**) 6-gingerol compared with the control (0 μM) on the viability of RAW264.7 cells for 24 h. Cell viability was determined using MTT assay (*n* = 3 independent experiments, *p* > 0.05, Kruskal–Wallis nonparametric test). Data represent the mean ± SEM.

**Figure 3:**
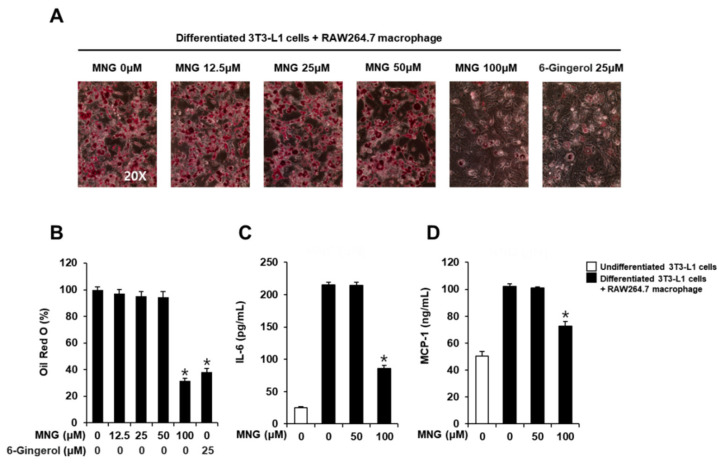
Inhibitory effects of the MNG on lipid accumulation and production of IL-6 and MCP-1 in co-culture conditions with 3T3-L1 and RAW264.7 cells. (**A**) Images of Oil Red O staining representing lipid accumulation and (**B**) levels of Oil Red O staining in co-culture condition with 3T3-L1 and RAW264.7 cells treated with the indicated concentration of MNG and 6-gingerol (positive control). Analysis of levels of the proinflammatory cytokine (**C**) IL-6 and (**D**) MCP-1 in co-culture conditions with 3T3-L1 and RAW264.7 cells treated with the indicated concentration of MNG and 6-gingerol (*n* = 3 independent experiments, * *p* < 0.05, Kruskal–Wallis nonparametric test). Data represent the mean ± SEM.

The cytotoxicity of MNG and 6-gingerol (positive control) was evaluated in 3T3-L1 preadipocytes and RAW264.7 cells, respectively. The cell viability assay showed that MNG up to a concentration of 100 µM and 6-gingerol up to a concentration of 25 µM did not affect the viability of 3T3-L1 preadipocytes and RAW264.7 cells, respectively (Figure 2). Thus, MNG (12.5, 25, 50, and 100 μM) and 6-gingerol (25 µM) were used in subsequent experiments using co-culture conditions with 3T3-L1 and RAW264.7 cells. In the co-culture with 3T3-L1 and RAW264.7 cells, 100 μM MNG and 25 µM 6-gingerol inhibited the levels of Oil Red O staining. The inhibitory effects of 100 μM MNG and 25 µM 6-gingerol were similar (Figure 3A,B). This result showed that MNG inhibited intracellular lipid accumulation in the co-culture system. In addition, 100 μM MNG inhibited IL-6 and MCP-1 production compared with the co-culture control (Figure 3C,D). This result indicated that MNG inhibited proinflammatory cytokine production in the co-culture system.

### 2.3. Inhibitory Effects of MNG on Adipogenesis- and Inflammation-Associated Proteins in Co-Culture Condition with 3T3-L1 and RAW264.7 Cells

**Figure 4:**
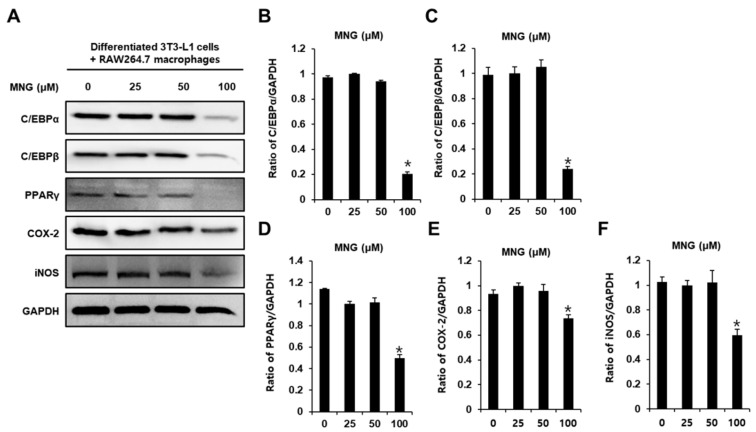
Inhibitory effects of MNG on adipogenesis- and inflammation-associated proteins in co-culture condition with 3T3-L1 and RAW264.7 cells. (**A**) Protein expression of CCAAT/enhancer-binding protein alpha (C/EBPα), C/EBPβ, peroxisome proliferator-activated receptor γ (PPARγ), cyclooxygenase 2 (COX-2), and inducible nitric oxide synthase (iNOS) in co-culture condition with 3T3-L1 and RAW264.7 cells treated with the indicated concentration of MNG. (**B**–**F**) Analysis of ratios of band intensities of C/EBPα, C/EBPβ, PPARγ, COX-2, and iNOS. (*n* = 3 independent experiments, * *p* < 0.05, Kruskal–Wallis nonparametric test). Data represent the mean ± SEM.

Western blotting was performed to investigate the protein expression of CCAAT/enhancer-binding protein alpha (C/EBPα), C/EBPβ, peroxisome proliferator-activated receptor γ (PPARγ), cyclooxygenase 2 (COX-2), and inducible nitric oxide synthase (iNOS). Consistent with the inhibition of intracellular lipid accumulation and proinflammatory cytokines, 100 μM MNG decreased the protein expression of C/EBPα, C/EBPβ, PPARγ, COX-2, and iNOS (Figure 4). These results indicated that MNG inhibited adipocyte-mediated inflammation.

## 3. Discussion

In this study, we investigated the effects of MNG on obesity-induced inflammatory responses in 3T3-L1 and RAW264.7 cells. MNG inhibited lipid accumulation in both 3T3-L1 and RAW264.7 cells. The inhibitory effects of 100 μM MNG and 25 µM 6-gingerol (the positive control) were similar. The compound 6-Gingerol, found in ginger, has been well reported for its anti-obesity effect [7,8,9]. Reportedly, 25 µM 6-gingerol inhibits lipid accumulation and obesity-induced inflammatory responses by inhibiting the production of the inflammatory mediators MCP-1 and IL-6 in co-culture conditions with 3T3-L1 and RAW264.7 cells [5]. In this study, consistent with the inhibition of intracellular lipid accumulation, MNG inhibited the production of the inflammatory mediators MCP-1 and IL-6 in co-culture conditions with 3T3-L1 and RAW264.7 cells. Studies have reported that obesity is associated with inflammation that contributes to impaired insulin signaling in adipose tissue, liver tissue, and skeletal muscle [10]. Obesity is characterized by an increase in macrophage infiltration into adipose tissue. Excessive lipid accumulation in adipocytes during obesity increases the availability of circulating fatty acids that stimulate macrophages. During the interaction between adipocytes and macrophages, excessive proinflammatory cytokines MCP-1 and IL-6 activate the inflammatory response [11]. Our results indicate that MNG inhibits obesity-induced excessive proinflammatory cytokines. In addition, MNG inhibited the protein expression of C/EBPβ, C/EBPα, PPARγ, COX-2, and iNOS in co-culture conditions with 3T3-L1 and RAW264.7 cells. In the molecular pathways related to adipogenesis and lipid accumulation, C/EBPβ, a nuclear transcription factor, activates C/EBPα and PPARγ to promote adipogenesis and lipid accumulation [12]. A study has demonstrated that an adipocyte-mediated increase in COX-2 and iNOS generates a large amount of NO, which leads to insulin resistance in adipose tissues [9,13]. Thus, our results demonstrated that MNG inhibited lipid accumulation and inflammatory response by downregulating IL-6 and MCP-1 production and protein expression of C/EBPβ, C/EBPα, PPARγ, COX-2, and iNOS in co-culture conditions with 3T3-L1 and RAW264.7 cells. These results suggest that MNG may be beneficial in preventing obesity-related inflammatory diseases. Additional reliable evidence from further research is necessary to evaluate whether MNG shows the same effects in animal models.

## 4. Materials and Methods

### 4.1. Extraction and Isolation

Jing Brand Liqueur (10 mL) was dried to yield a sample (1.66 g), which was then dissolved in 10 mL of water. The aqueous solution was loaded onto an open column (HP20 6.6 g, 1.5 × 6.0 cm). A gradient solvent system from 100% water to 100% methanol (0, 20, 50, 80, and 100% MeOH/H_2_O) was used for HP20 open column separation, and the eluents were dried using SpeedVac to yield five fractions (Fr. I: 1.5 g; Fr. II: 134 mg; Fr. III: 93.0 mg; Fr. IV: 8.0 mg; Fr. V: 1.3 mg). Fraction V was separated with a Thermo Scientific Ultimate 3000 preparative HPLC system (Thermo Fisher Scientific, Sunnyvale, CA, USA) (Column: Phenomenex Luna C18, 100 Å, 100 × 21.2 mm, 5 μm; flow rate: 10 mL/min) and then a Thermo Scientific Ultimate 3000 semi-preparative HPLC system (Column: Phenomenex Luna C18 or C8, 100 Å, 250 × 10 mm, 5 μm; flow rate: 3 mL/min) to obtain pure MNG. In order to obtain a sufficient amount of MNG for structure determination, 160 L Jing Brand liqueur was dried, and approximately 0.5 mg of MNG (3.1 g/L) was obtained [7,8]. For the biological experiments, MNG (#QP-1913) was purchased from Quality Phytochemicals LLC (East Brunswick, NJ, USA).

### 4.2. Cell Culture

The murine preadipocyte (3T3-L1) and murine macrophage (RAW264.7) lines were purchased from the American Type Culture Collection (Manassas, VA, USA). The 3T3-L1 preadipocytes were grown in Dulbecco’s modified Eagle’s medium (DMEM) (Cellgro, Manassas, VA, USA) supplemented with 1% penicillin/streptomycin (P/S) and 10% fetal calf serum (Invitrogen Co., Grand Island, NY, USA) at 37 °C in 5% CO_2_ and 95% humidity. RAW264.7 cells were grown in DMEM supplemented with 1% P/S and 10% fetal bovine serum (Invitrogen Co., Grand Island, NY, USA) at 37 °C in 5% CO_2_ and 95% humidity.

### 4.3. Cell Viability Assays

The nontoxic concentration ranges of MNG in 3T3-L1 preadipocytes and RAW264.7 cells were evaluated using an Ez-Cytox cell viability assay kit (Daeil Lab Service Co., Seoul, Korea). Cells were seeded and incubated with various concentrations of MNG for 24 h. Cell viability was measured using a microplate reader (PowerWave XS; Bio-Tek Instruments, Winooski, VT, USA) at 450 nm after reaction for 1 h at 37 °C, followed by the addition of 10% (*v*/*v*) Ez-Cytox reagent.

### 4.4. Adipogenic Differentiation Assays and Co-Culture

The 3T3-L1 preadipocytes were seeded and stimulated with 0.5 mM 1-isobutyl-3-methylxanthine, 5 µg/mL insulin, and 1 µg/mL dexamethasone for 2 days. The culture medium was replenished with 5 µg/mL of insulin for 2 days. The culture medium was replaced every 2 days until day 8. On day 8, differentiated adipocytes were co-cultured with RAW264.7 cells.

### 4.5. Oil Red O Staining

Differentiated 3T3-L1 adipocytes co-cultured with RAW264.7 cells were treated with MNG, fixed with 4% paraformaldehyde solution for 1 h, and stained with Oil Red O solution for 1 h. Lipid accumulation was observed using a light microscope at a magnification of 20×. The plates were treated with 100% isopropanol, and the optical density of the eluted Oil Red O solution at 520 nm was determined using a microplate reader (PowerWave XS; Bio-Tek Instruments, Winooski, VT, USA).

### 4.6. Measurement of Proinflammatory Cytokine Levels

Cell culture supernatants were collected and assayed using ELISA kits specific for MCP-1 and IL-6, according to the manufacturer’s protocol (R&D Systems, Minneapolis, MN, USA). The levels of MCP-1 and IL-6 were determined by measuring absorbance at 450 nm using a microplate reader (PowerWave XS; Bio-Tek Instruments, Winooski, VT, USA).

### 4.7. Western Blot Analysis

Differentiated 3T3-L1 adipocytes co-cultured with RAW264.7 cells were treated with MNG and lysed using RIPA buffer (Cell Signaling, Danvers, MA, USA). Equal amounts of protein extracts (20 μg) were separated on a 10% sodium dodecyl sulfate-polyacrylamide gel and transferred onto polyvinylidene difluoride membranes. The membranes were incubated with primary antibodies (1:1000; Cell Signaling Technology, Inc., Danvers, MA, USA) against C/EBPα, C/EBPβ, PPARγ, COX-2, and iNOS overnight at 4 °C, followed by incubation with secondary antibodies (1:2000; Cell Signaling Technology, Inc.). ECL Plus Western blotting detection reagent (GE Healthcare, Little Chalfont, UK) was used, and the signals were visualized using a chemiluminescence system (FUSION Solo, PEQLAB Biotechnologie GmbH, Erlangen, Germany).

### 4.8. Statistical Analysis

All experiments were performed in triplicate. All analyses were performed using SPSS Statistics ver. 19.0 (SPSS Inc., Chicago, IL, USA). Nonparametric comparisons of samples were conducted using the Kruskal–Wallis test to analyze the results. Differences were considered statistically significant at *p* < 0.05.

## 5. Conclusions

In the co-culture condition with 3T3-L1 and RAW264.7 cells, MNG inhibited lipid accumulation and production of IL-6 and MCP-1. Moreover, MNG inhibited adipogenesis- and inflammation-associated proteins, including C/EBPα, C/EBPβ, PPARγ, COX-2, and iNOS, in co-culture conditions with 3T3-L1 and RAW264.7 cells. These findings provide evidence that MNG inhibits adipocyte-mediated inflammation and that MNG is a potential dietary source that has an inhibitory effect on obesity-induced inflammation.

## References

[1] Matsuda M., Shimomura I. (2013). Increased oxidative stress in obesity: Implications for metabolic syndrome, diabetes, hypertension, dyslipidemia, atherosclerosis, and cancer. Obes. Res. Clin. Pract..

[2] Kamei N., Tobe K., Suzuki R., Ohsugi M., Watanabe T., Kubota N., Ohtsuka-Kowatari N., Kumagai K., Sakamoto K., Kobayashi M. (2006). Overexpression of monocyte chemoattractant protein-1 in adipose tissues causes macrophage recruitment and insulin resistance. J. Biol. Chem..

[3] Makki K., Froguel P., Wolowczuk I. (2013). Adipose tissue in obesity-related inflammation and insulin resistance: Cells, cytokines, and chemokines. Int. Sch. Res. Not..

[4] Kim C.Y., Kang B., Suh H.J., Choi H.-S. (2018). Red ginseng-derived saponin fraction suppresses the obesity-induced inflammatory responses via Nrf2-HO-1 pathway in adipocyte-macrophage co-culture system. Biomed. Pharmacother..

[5] Choi J., Kim K.-J., Kim B.-H., Koh E.-J., Seo M.-J., Lee B.-Y. (2017). 6-Gingerol suppresses adipocyte-derived mediators of inflammation in vitro and in high-fat diet-induced obese zebra fish. Planta Med..

[6] Hsieh C.-C., Chou M.-J., Wang C.-H. (2017). Lunasin attenuates obesity-related inflammation in RAW264. 7 cells and 3T3-L1 adipocytes by inhibiting inflammatory cytokine production. PLoS ONE.

[7] Cai Y., Xu J., Chen M., Wang D., Yang Y., Manavalan A., Wu X., Liu Y., Cao S. (2020). Compound Analysis of Jing Liqueur and Nrf2 Activation by Jing Liqueur–One of the Most Popular Beverages in China. Beverages.

[8] Wu X., Xu J., Cai Y., Yang Y., Liu Y., Cao S. (2021). Cytoprotection against Oxidative Stress by methylnis-solin-3-O-β-D-glucopyranoside from *Astragalus membranaceus* mainly via the Activation of the Nrf2/HO-1 Pathway. Molecules.

[9] Tzeng T.-F., Liu I.-M. (2013). 6-Gingerol prevents adipogenesis and the accumulation of cytoplasmic lipid droplets in 3T3-L1 cells. Phytomedicine.

[10] Tzeng T.F., Chang C.J., Liu I.M. (2014). 6-Gingerol inhibits rosiglitazone-induced adipogenesis in 3T3-L1 adipocytes. Phytother. Res..

[11] Isa Y., Miyakawa Y., Yanagisawa M., Goto T., Kang M.-S., Kawada T., Morimitsu Y., Kubota K., Tsuda T. (2008). 6-Shogaol and 6-gingerol, the pungent of ginger, inhibit TNF-α mediated downregulation of adiponectin expression via different mechanisms in 3T3-L1 adipocytes. Biochem. Biophys. Res..

[12] He F., Huang Y., Song Z., Zhou H.J., Zhang H., Perry R.J., Shulman G.I., Min W. (2021). Mitophagy-mediated adipose inflammation contributes to type 2 diabetes with hepatic insulin resistance. J. Exp. Med..

[13] DeOliveira C.C., Acedo S.C., Gotardo É.M.F., de Oliveira Carvalho P., Rocha T., Pedrazzoli J., Gambero A. (2012). Effects of methotrexate on inflammatory alterations induced by obesity: An in vivo and in vitro study. Mol. Cell. Endocrinol..

